# Supplementary material for: Graphene-based autonomous pyroelectric system for near-field energy conversion
Source: Sci Rep. 2021 Sep 30;11:19489. doi: 10.1038/s41598-021-98656-8 (PMC8484632; doi:10.1038/s41598-021-98656-8)
Supplement: Supplementary file 1 — Supplementary Information. [file 41598_2021_98656_MOESM1_ESM.pdf]

Supplementary Information

Graphene-based autonomous pyrolectric system for near-field energy conversion

Ivan Latella and Philippe Ben-Abdallah

Supplementary Section 1. Volumetric heat capacity of the active layer

The active zone is a multilayer structure consisting of the pyroelectric material of thickness δ_p covered by Au layers acting as electrodes and superficial layers of SiO_2. Denoting by δ_e and δ_s the total thicknesses of the electrodes and superficial layers, respectively, the volumetric heat capacity c_v of the active zone with overall thickness δ is defined by

$$c_v \equiv \frac{1}{\delta}(c_e\delta_e + c_s\delta_s + c_p\delta_p), \tag{1}$$

where c_e, c_s and c_p are the specific heat capacities per unit volume of the electrodes, superficial layers and pyroelectric material, respectively. For the Au electrodes we take $c_e = 2.4\times10^6\,\mathrm{J\,m^{-3}\,K^{-1}}$, while for the SiO_2 layes we assume $c_s = 1.48\times10^6\,\mathrm{J\,m^{-3}\,K^{-1}}$. The heat capacities c_p of the considered pyroelectric materials are given in the main text, except that for $BaTiO_3$ which is given below.

Supplementary Section 2. Temperature dependent properties of barium titanate

We fit the specific heat of the $BaTiO_3$ film from experimental data coming from ac-hot probe measurements on thin films deposited on massive substrates[1]. The specific heat (in $\mathrm{J\,mol^{-1}\,K^{-1}}$) for a 100 m thick film of this material follows the law

$$C_{\mathrm{BaTiO_3}} = \begin{cases} 110.4 & \text{if } T < 360 \\ 0.002(T-360)^{2.5} + 110.4 & \text{if } 360 < T < 387.6 \\ 0.000685(395-T)^{4.5} + 112.9 & \text{if } 387.6 < T < 395 \\ 112.9 & \text{if } T > 395 \end{cases} \tag{2}$$

with T in kelvin. For this material, the molar mass is $m_{\mathrm{BaTiO_3}} = 233.192\,\mathrm{g\,mol^{-1}}$, the denisty $\rho_{\mathrm{BaTiO_3}} = 6.02\times10^6\,\mathrm{g\,m^{-3}}$ and thus the volumetric heat capacity is $c_p = \rho_{\mathrm{BaTiO_3}}C_{\mathrm{BaTiO_3}}/m_{\mathrm{BaTiO_3}}$ (in $\mathrm{J\,m^{-3}\,K^{-1}}$).

The pyroelectric coefficient p of $BaTiO_3$ ceramic used for the calculations in the main text is shown in the Supplementary Figure 1 as a function of the temperature. It corresponds to experimental measurements by Lang et al.[2] obtained with a dynamic method. In the inset of this figure we show the permittivity[3] ε_{33} of $BaTiO_3$ in units of the vacuum permittivity ε_0.

Supplementary Section 3. Synchronized electric charge extraction (SECE) cycle

The SECE cycle has been described in detail by Sebald et al.[4] and here we repeat their analysis by explicitly including the temperature dependence of the pyroelectric coefficient p. The equations for displacement D and entropy density S (per unit volume) of the ferroelectric material are

$$\begin{aligned} dD &= \varepsilon_{33}dE + pdT, \\ TdS &= TpdE + c_p dT, \end{aligned} \tag{3}$$

where E is the electric field, T is the temperature, ε_{33} is the permittivity and c_p is the specific heat of the material at constant field. The SECE cycle consist of the following four steps:

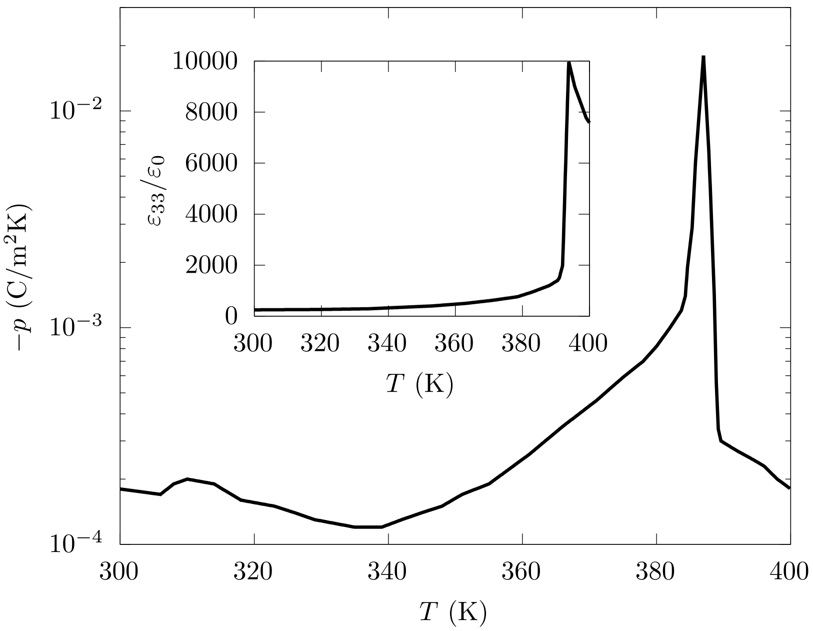

Supplementary Figure 1. Pyroelectric coefficient and permittivity of $BaTiO_3$ as a function of the temperature.

1) Open-circuit heating from $T_{\min}$ to $T_{\max}$. The open-circuit field, initially at $E=0$, reaches the maximum value $E_{\max}$.

2) Isothermal electric charge extraction: the field is decreased from $E_{\max}$ to 0 at temperature $T_{\max}$.

3) Open-circuit cooling from $T_{\max}$ to $T_{\min}$. The open-circuit field, initially at $E=0$, reaches the minimum value $E_{\min}$.

4) Isothermal electric charge extraction: the field is increased from $E_{\min}$ to 0 at temperature $T_{\min}$.

During step 1), the displacement remains constant, so that the open-circuit field changes with the temperature as $dE=-p/\varepsilon_{33}\,dT$. The maximum value of the field achieved in this step is then given by

$$E_{\max}=-\int_{T_{\min}}^{T_{\max}}\frac{p}{\varepsilon_{33}}dT. \tag{4}$$

Under these conditions, the equation for the entropy variation becomes $TdS=-Tp^2/\varepsilon_{33}\,dT+c_p\,dT$, and the heat per unit volume absorbed by the material $Q_1=\int_{T_{\min}}^{T_{\max}}TdS$ is given by

$$Q_1=\int_{T_{\min}}^{T_{\max}}c_p dT-\int_{T_{\min}}^{T_{\max}}\frac{p^2}{\varepsilon_{33}}TdT. \tag{5}$$

During step 2), the field is decreased isothermally and the variation of entropy is given by $dS=p\,dE$ which after integration gives $\Delta S_2=-p(T_{\max})E_{\max}$, where we have assumed that the pyroelectric coefficient does not depend on the field. The heat absorbed during this step is $Q_2=T_{\max}\Delta S_2$ and can be written as

$$Q_2=T_{\max}p(T_{\max})\int_{T_{\min}}^{T_{\max}}\frac{p}{\varepsilon_{33}}dT, \tag{6}$$

where we have expressed $E_{\max}$ using Eq. (4). For step 3), repeating the procedure in step 1), one obtains

$$Q_3=-\int_{T_{\min}}^{T_{\max}}c_p dT+\int_{T_{\min}}^{T_{\max}}\frac{p^2}{\varepsilon_{33}}TdT, \tag{7}$$

while for step 4), which is analogous to step 2), one gets

$$Q_4 = -T_{\rm min} p(T_{\rm min}) \int_{T_{\rm min}}^{T_{\rm max}} \frac{p}{\varepsilon_{33}} dT. \tag{8}$$

Thus, summing up the heat absorbed in the whole cycle, $Q = \sum_i Q_i$, yields

$$Q = [T_{\rm max} p(T_{\rm max}) - T_{\rm min} p(T_{\rm min})] \int_{T_{\rm min}}^{T_{\rm max}} \frac{p}{\varepsilon_{33}} dT. \tag{9}$$

According to the first law, the variation of internal energy is given by $\Delta U = Q - W$, where W is the work per unit volume delivered by the system. Since in a cycle we have $\Delta U = 0$, the delivered work per unit surface is

$$W_p = \delta_p [T_{\rm max} p(T_{\rm max}) - T_{\rm min} p(T_{\rm min})] \int_{T_{\rm min}}^{T_{\rm max}} \frac{p}{\varepsilon_{33}} dT, \tag{10}$$

where we have multiplied by the thickness of the material δ_p. Furthermore, the total energy input is given by the heat absorbed during steps 1 and 2, $Q_h = Q_1 + Q_2$, which is given by

$$Q_h = \int_{T_{\rm min}}^{T_{\rm max}} c_p dT + T_{\rm max} p(T_{\rm max}) \int_{T_{\rm min}}^{T_{\rm max}} \frac{p}{\varepsilon_{33}} dT - \int_{T_{\rm min}}^{T_{\rm max}} \frac{p^2}{\varepsilon_{33}} T dT. \tag{11}$$

The second and third term above are typically small as compared to the first one and can be neglected for most materials, so $Q_h \approx \int_{T_{\rm min}}^{T_{\rm max}} c_p dT$ on the pyroelectric material. Noting also that the other layers in the active zone absorb heat during the cycle, the energy input per unit surface takes the form $W_{\rm in} = \delta \int_{T_{\rm min}}^{T_{\rm max}} c_v(T_2)\, dT_2$, where c_v is the heat capacity of the active zone and δ its total thickness. We point out that the heat absorption during the temperature variation follows also from time integration of the radiative energy flux absorbed by the membrane, $\delta \int_{T_{\rm min}}^{T_{\rm max}} c_v(T_2)\, dT_2 = \int \mathcal{P}_r(t)\, dt$, where the integration time interval corresponds to the heating stage of the cycle.

Supplementary Section 4. Ericsson cycle

The Ericsson cycle consist of two isothermal and two constant electric field paths[5] and has been extensively considered for pyroelectric energy conversion[6]. For completeness, here we include a brief thermodynamic description of this cycle following the work by Sebald et al.[7]

The steps of the cycle can be listed as follows:

1) At constant temperature $T_{\rm min}$, the applied field increases from $E_{\rm min}$ to $E_{\rm max}$.

2) At constant field $E_{\rm max}$, the temperature increases from $T_{\rm min}$ to $T_{\rm max}$.

3) At constant temperature $T_{\rm max}$, the applied field decreases from $E_{\rm max}$ to $E_{\rm min}$.

4) At constant field $E_{\rm min}$, the temperature decreases from $T_{\rm max}$ to $T_{\rm min}$.

As in the previous cycle, the starting point is the thermodynamic characterization given by Eqs. (3). By integrating the TdS equation, the heat Q_i absorbed by the material in each step reads

$$Q_1 = T_{\rm min} \int_{E_{\rm min}}^{E_{\rm max}} p(E, T_{\rm min})\, dE, \tag{12}$$

$$Q_2 = \int_{T_{\rm min}}^{T_{\rm max}} c_p(E_{\rm max}, T)\, dT, \tag{13}$$

$$Q_3 = T_{\rm max} \int_{E_{\rm max}}^{E_{\rm min}} p(E, T_{\rm max})\, dE, \tag{14}$$

$$Q_4 = \int_{T_{\rm max}}^{T_{\rm min}} c_p(E_{\rm min}, T)\, dT. \tag{15}$$

Hence, neglecting the temperature dependence of p in the working temperature range, assuming that the heat capacity does not depend on the applied field and taking $E_{\text{min}} = 0$, the sum of the heat for the whole cycle $Q = \sum_i Q_i$ is given by $Q = -(T_{\text{max}} - T_{\text{min}}) \int_0^{E_{\text{max}}} p(E)\, dE$. Because $\Delta U = Q - W$ and $\Delta U = 0$ in a cycle, the delivered work per unit surface is

$$W_p = \delta_p (T_{\text{max}} - T_{\text{min}}) \int_0^{E_{\text{max}}} p(E)\, dE. \tag{16}$$

Finally, the energy taken by the material to increase its temperature and at the high temperature isotherm is $Q_h = Q_2 + Q_3 = \int_{T_{\text{min}}}^{T_{\text{max}}} c_p(T)\, dT + T_{\text{max}} \int_0^{E_{\text{max}}} p(E)\, dE$. Multiplying this quantity by the thickness of the pyroelectric material and adding the energy absorved by the other layers in the active zone, the input energy per unit surface is given by

$$W_{\text{in}} = \delta \int_{T_{\text{min}}}^{T_{\text{max}}} c_v(T)\, dT + \delta_p T_{\text{max}} \int_0^{E_{\text{max}}} p(E)\, dE. \tag{17}$$

References

1. B. A. Strukov, S. T. Davitadze, S. N. Kravchun, S. A. Taraskin, B. M. Goltzman, V. V. Lemanov, S. G. Shulman, Specific heat and heat conductivity of the BaTiO3 polycrystalline films with the thickness in the range 20 - 1100 nm, J.Phys.: Condens. Matter, 15, 4331-4340, (2003).
2. S. B. Lang, L. H. Rice and S. A. Shaw, Pyroelectric Effect in Barium Titanate Ceramic, J. Appl. Phys. 40, 4335 (1969).
3. Merz, W. J. The electric and optical behavior of $BaTiO_3$ single-domain crystals. Phys. Rev. **76**, 1221 (1949).
4. G. Sebald, E. Lefeuvre and D.l Guyomar, Pyroelectric Energy Conversion: Optimization principles, IEEE Trans. Ultrason. Ferroelectr. Freq. Control 55, 538-551 (2008).
5. R. B. Olsen, D. A. Bruno and J. M. Briscoe, Pyroelectric conversion cycles, J. Appl. Phys. 58, 4709-4716 (1985).
6. C. R. Bowen, J. Taylor, E. LeBoulbar, D. Zabek, A. Chauhanc and R. Vaish, Pyroelectric materials and devices for energy harvesting applications, Energy Environ. Sci., 7, 3836 (2014).
7. G. Sebald, S. Pruvost and D. Guyomar, Energy Harvesting Based on Ericsson Pyroelectric Cycles in a Relaxor Ferroelectric Ceramic, Smart Mater. Struct., 17(1), 015012 (2008).
